# A teaching learning based optimization based on orthogonal design for solving global optimization problems

**DOI:** 10.1186/2193-1801-2-130

**Published:** 2013-03-23

**Authors:** Suresh Chandra Satapathy, Anima Naik, K Parvathi

**Affiliations:** ANITS, Vishakapatnam, India; MITS, Rayagada, India; CUTM, Paralakhemundi, India

**Keywords:** TLBO, Global function Optimization, Orthogonal design, Convergence speed

## Abstract

In searching for optimal solutions, teaching learning based optimization (TLBO) (Rao et al. 2011a; Rao et al. 2012; Rao & Savsani 2012a) algorithms, has been shown powerful. This paper presents an, improved version of TLBO algorithm based on orthogonal design, and we call it OTLBO (Orthogonal Teaching Learning Based Optimization). OTLBO makes TLBO faster and more robust. It uses orthogonal design and generates an optimal offspring by a statistical optimal method. A new selection strategy is applied to decrease the number of generations and make the algorithm converge faster. We evaluate OTLBO to solve some benchmark function optimization problems with a large number of local minima. Simulations indicate that OTLBO is able to find the near-optimal solutions in all cases. Compared to other state-of-the-art evolutionary algorithms, OTLBO performs significantly better in terms of the quality, speed, and stability of the final solutions.

## Introduction

Teaching-Learning based Optimization (TLBO) algorithm is a global optimization method originally developed by Rao et al. (Rao et al. 2011a; Rao et al. 2012; Rao & Savsani 2012a). It is a population- based iterative learning algorithm that exhibits some common characteristics with other evolutionary computation (EC) algorithms (Fogel 1995). However, TLBO searches for an optimum through each learner trying to achieve the experience of the teacher, which is treated as the most learned person in the society, thereby obtaining the optimum results, rather than through learners undergoing genetic operations like selection, crossover, and mutation (Shi & Eberhart 1998). Due to its simple concept and high efficiency, TLBO has become a very attractive optimization technique and has been successfully applied to many real world problems (Rao et al. 2011a; Rao et al. 2012; Rao & Savsani 2012a), (Rao et al. 2011b; Rao & Patel 2012; Rao & Savsani 2012b; Vedat 2012; Rao & Kalyankar 2012; Suresh Chandra & Anima 2011).

In any evolutionary algorithms the convergence rate is given prime importance for solving an optimization problem over quality of solutions. TLBO in general produces improved results in compared to other EC techniques like Genetic Algorithm (GA), Particle Swarm Optimization (PSO), Differential Evolution (DE), and Artificial Bee Colony (ABC)(Suresh Chandra et al. 2012). However, in real- world real time applications, the major thrust is always on convergence time. To make TLBO suitable for such applications, this work focuses on improving the convergence time while without compromising the quality of results.

In our proposed work, the attempt is made to include orthogonal design method in the basic TLBO optimization. In our approach, each learner in the class of learners can be divided into several partial vectors where each of them acts as a factor in the orthogonal design. Orthogonal design is then employed to search the best scales among all the various combinations. Orthogonal design method (Fang & Ma 2001) with both orthogonal array (OA) and factor analysis (such as the statistical optimal method) is developed to sample a small, but representative set of combinations for experimentation to obtain good combinations. OA is a fractional factorial array of numbers arranged in rows and columns, where each row represents the levels of factors in each combination, and each column represents a specific factor that can be changed from each combination. It can assure a balanced comparison of levels of any factor. The term “main effect” designates the effect on response variables that one can trace to a design parameter. The array is called orthogonal because all columns can be evaluated independently of one another, and the main effect of one factor does not bother the estimation of the main effect of another factor. Recently, some researchers applied the orthogonal design method incorporated with EAs to solve optimization problems. Leung and Wang (Leung & Wang 2001) incorporated orthogonal design in genetic algorithm for numerical optimization problems found such method was more robust and statistically sound. This method was also adopted by other researchers (Ding et al. 1997; Kui-fan et al. 2002; San-You et al. 2005; Wang et al. 2007; Wang et al. 2012) to solve optimization problems. Numerical results demonstrated that these techniques had a significantly better performance than the traditional EAs on the problems studied, and the resulting algorithm can be more robust and statistically sound. In this paper, the orthogonal design is implemented on TLBO (hence called OTLBO) to make it faster and more robust. It is shown empirically that OTLBO has high performance in solving benchmark functions comprising many parameters, as compared with some existing EAs.

The rest of this paper is organized as follows. “Teaching–learning-based optimization” briefly describes TLBO as a function optimization technique and “Orthogonal design” presents some properties of the orthogonal design method. “Proposed orthogonal teaching–learning-based optimizer (OTLBO)” presents the proposed OTLBO. In “Experimental results”, we test our algorithm through some benchmark functions which is followed by discussions and analysis of the optimization experiments for the OTLBO. The last section, “Conclusions and further study”, is devoted to conclusions and future studies.

## Teaching–learning-based optimization

This optimization method is based on the effect of the influence of a teacher on the output of learners in a class. It is a population based method and like other population based methods it uses a population of solutions to proceed to the global solution. A group of learners constitute the population in TLBO. In any optimization algorithms there are numbers of different design variables. The different design variables in TLBO are analogous to different subjects offered to learners and the learners’ result is analogous to the ‘fitness’, as in other population-based optimization techniques. As the teacher is considered the most learned person in the society, the best solution so far is analogous to Teacher in TLBO. The process of TLBO is divided into two parts. The first part consists of the “Teacher phase” and the second part consists of the “Learner phase”. The “Teacher phase” means learning from the teacher and the “Learner phase” means learning through the interaction between learners. In the sub-sections below we briefly discuss the implementation of TLBO.

### Initialization

Following are the notations used for describing the TLBO

*N*: number of learners in class i.e. “class size”

*D*: number of courses offered to the learners

*MAXIT*: maximum number of allowable iterations

The population *X* is randomly initialized by a search space bounded by matrix of *N* rows and *D* columns. The *jth* parameter of the *ith* learner is assigned values randomly using the equation

1 $$x_{\left(i,j\right)}^{0}=x_{j}^{\min}+\mathrm{rand}\times \left(x_{j}^{\max}-x_{j}^{\min}\right)$$

where *rand* represents a uniformly distributed random variable within the range (0, 1), $\;x_{j}^{\min}$ and $x_{j}^{\max\;}$ represent the minimum and maximum value for *jth* parameter. The parameters of *ith* learner for the generation *g* are given by

2 $$\;X_{\left(i\right)}^{g}=\left[x_{\left(i,1\right)}^{g},x_{\left(i,2\right)}^{g},x_{\left(i,3\right)}^{g},\ldots \ldots ,x_{\left(i,j\right)}^{g},\ldots \ldots ,x_{\left(i,D\right)}^{g}\right]$$

### Teacher phase

The mean parameter *M*^*g*^ of each subject of the learners in the class at generation *g* is given as

3 $$M^{g}=\left[m_{1}^{g},m_{2}^{g},\ldots \ldots ,m_{j}^{g},\ldots \ldots ,m_{D}^{g}\right]$$

The learner with the minimum objective function value is considered as the teacher $X_{\mathrm{Teacher}}^{g}$ for respective iteration. The Teacher phase makes the algorithm proceed by shifting the mean of the learners towards its teacher. To obtain a new set of improved learners a random weighted differential vector is formed from the current mean and the desired mean parameters and added to the existing population of learners.

4 $$\;\mathrm{Xne}w_{\left(i\right)}^{g}=X_{\left(i\right)}^{g}+\mathrm{rand}\times \left(X_{\mathrm{Teacher}}^{g}-T_{F}M^{g}\right)$$

*T*_*F*_ is the teaching factor which decides the value of mean to be changed. Value of *T*_*F*_ can be either 1 or 2. The value of *T*_*F*_ is decided randomly with equal probability as,

5 $$T_{F}=\mathrm{round}\;\left[1+\mathrm{rand}\left(0,1\right)\left\{2–1\right\}\right]$$

Where  *T*_*F*_ is not a parameter of the TLBO algorithm. The value of *T*_*F*_ is not given as an input to the algorithm and its value is randomly decided by the algorithm using Eq. (5). After conducting a number of experiments on many benchmark functions it is concluded that the algorithm performs better if the value of *T*_*F*_ is between 1 and 2. However, the algorithm is found to perform much better if the value of *T*_*F*_ is either 1 or 2 and hence to simplify the algorithm, the teaching factor is suggested to take either 1 or 2 depending on the rounding up criteria given by Eq. (5).

If $\mathrm{Xne}w_{\left(i\right)}^{g}$ is found to be a superior learner than $X_{\left(i\right)}^{g}$ in generation *g* , than it replaces inferior learner $X_{\left(i\right)}^{g}$ in the matrix.

### Learner phase

In this phase the interaction of learners with one another takes place. The process of mutual interaction tends to increase the knowledge of the learner. The random interaction among learners improves his or her knowledge. For a given learner $X_{\left(i\right)}^{g}$, another learner $X_{\left(r\right)}^{g}$ is randomly selected (*i* ≠ *r*). The *ith* parameter of the matrix *Xnew* in the learner phase is given as

6 $$\mathrm{Xne}w_{\left(i\right)}^{g}=\left\{\begin{array}{c} X_{\left(i\right)}^{g}+\mathrm{rand}\times \left(X_{\left(i\right)}^{g}-X_{\left(r\right)}^{g}\right)\;\mathrm{if}\;f\left(X_{\left(i\right)}^{g}\right)<f\left(X_{\left(r\right)}^{g}\right)\\ X_{\left(i\right)}^{g}+\mathrm{rand}\times \left(X_{\left(r\right)}^{g}-X_{\left(i\right)}^{g}\right)\;\mathrm{oterwise} \end{array}\right.$$

### Algorithm termination

The algorithm is terminated after *MAXIT* iterations are completed.

Details of TLBO can be refereed in (Rao et al. 2011a; Rao et al. 2012; Rao & Savsani 2012a).

## Orthogonal design

Consider an experiment that involves some *factors*, each of which have several possible values called *levels*. Suppose that there are P factors, each factor has Q levels. The number of combinations is *Q*^*P*^, and for large P and Q it is not practical to evaluate all combinations.

*Orthogonal design* has been developed as a mathematical tool to study multi-factor and multi-level problems. It aims to extract an orthogonal array L of M rows, where each row represents a combination to be evaluated. The array has three key properties.

1. During the experiment, the array represents a subset of M combinations, from all possible *Q*^*P*^ combinations. Computation is reduced considerably because M << *Q*^*P*^*.*
2. Each column represents a factor. If some columns are deleted from the array, it means a smaller number of factors are considered.
3. The columns of the array are orthogonal to each other. The selected subset is scattered uniformly over the search space to ensure its diversity.

A simple but efficient method is proposed in (Wing-Leung & Yuping 2001) to generate an orthogonal array L where M = Q×Q and P = Q + 1. The steps of this method are shown in Algorithm 1.

## Proposed orthogonal teaching–learning-based

Algorithm 1: Procedure for generating an orthogonal array L.

## Proposed orthogonal teaching–learning-based optimizer (OTLBO)

We propose a teaching learning based optimization approach based on orthogonal design (OD). In our approach, termed OTLBO, each learner in the class of learners can be divided into several partial vectors where each of them acts as a factor in the orthogonal design. Orthogonal design is then employed to search the best scales among all the various combinations. Compared to previous OD-based methods (Wing-Leung & Yuping 2001; Wenyin et al. 2006; Shinn-Ying et al. 2008; Sanyou et al. 2007; Kwon-Hee et al. 2003), our proposed algorithm has the following:

### OD-based operator and updating strategy

Each solution to the optimization problem is represented as a learner. For an objective function with N variables, a learner is encoded in the form of

$$X_{i}=\left[x_{i,1},x_{i,2},x_{i,3},\ldots ,x_{i,N}\right],i=1,2,3,\ldots \ldots \ldots ,S$$

where S is the population size.

The standard TLBO algorithm updates the current learner by comparing with best learner (i.e. teacher) in teacher phase and with a randomly select learner in learner phase. It lacks the interaction between neighboring learners and it may easily trapped into local minima. One technique to address this problem is to employ the multi-parent crossover during evolution and this technique has been shown to improve the convergence rate when applied to GAs.

Given m learners, the question is how to execute the multi-parent efficiently. Since each learner consists of N factors, there are *m*^*N*^ combinations. Consequently, the orthogonal design method is employed to select m (if a  *L*_*m*_ (*Q*^*P*^ ) orthogonal array is considered, where P = N and Q = m) representative sets of combinations to shorten the computational time. The procedure of OD-based multi-parent is detailed in Algorithm 2.

Algorithm 2: OD-based operator for m learners. 

Compute the fitness for all *р*_*i*, *j*_.

Mix *р*_*i*, *j*_ and *X*_*i*, *j*_ and rank learners in the decreasing order of fitness

Select the top m learners as the output.

*Remark 1:* The OD-based operator behaves as the local search among the selected learners.

### Steps of OD-based TLBO

To obtain a more precise solution compared to the standard TLBO, the OD-based operator is employed. The elitism preservation strategy for upgrading the current population is proposed, in which the learner is updated only if its fitness is improved. The procedure for the OD-based TLBO is shown in Algorithm 3. A convergence criterion or the maximum run can be used as the termination condition.

Algorithm 3: OD-based TLBOAlgorithm 3: OD-based TLBO

Take the learner g as the output. *Remark 2:* The convergence of OTLBO is guaranteed because of the elitism preservation strategy. A learner moves only if the movement will lower this objective function.

## Experimental results

We have divided our experimental works into four parts. In part1, we have done performance comparison of OTLBO against basic algorithms like PSO, DE and TLBO to establish that our proposed approach performs better than above algorithms for investigated problems. We used 20 benchmark problems in order to test the performance of the PSO, DE, TLBO and the OTLBO algorithms. This set is large enough to include many different kinds of problems such as unimodal, multimodal, regular, irregular, separable, non-separable and multidimensional. For experiments in part2 to part4 few functions from these 20 functions are used and those are mentioned in the comparison tables in respective sections of the paper. Initial range, formulation, characteristics and the dimensions of these problems are listed in Table 1. Also we have used Friedman’s test to determine the average ranking of algorithms based on their performance in each part.

**Table 1:** **Benchmark functions,** ***D*** **Dimension,** ***C*** **Characteristic,** ***U*** **Unimodal,** ***M*** **Multimodal,** ***S*** **Separable,** ***N*** **Non-separable**

No.	Function	D	C	Range	Formulation	Value
*f*_1_	Step	30	US	[−100,100]	$f\left(x\right)=\overset{D}{\underset{i=1}{\sum }}{\left(x_{i}+0.5\right)}^{2}$	*f*_*min*_=0
*f*_2_	Sphere	30	US	[−100,100]	$f\left(x\right)=\overset{D}{\underset{i=1}{\sum }}x_{i}^{2}$	*f*_*min*_=0
*f*_3_	SumSquares	30	US	[−100,100]	$f\left(x\right)=\overset{D}{\underset{i=1}{\sum }}ix_{i}^{2}$	*f*_*min*_=0
*f*_4_	Quartic	30	US	[−1.28,1.28]	$f\left(x\right)=\overset{D}{\underset{i=1}{\sum }}ix_{i}^{4}+\mathrm{random}\left(0,1\right)$	*f*_*min*_=0
*f*_5_	Zakharov	10	UN	[−5,10]	$f\left(x\right)=\overset{D}{\underset{i=1}{\sum }}x_{i}^{2}+{\left(\overset{D}{\underset{i=1}{\sum }}0.5ix_{i}\right)}^{2}+{\left(\overset{D}{\underset{i=1}{\sum }}0.5ix_{i}\right)}^{4}$	*f*_*min*_=0
*f*_6_	Schwefel 1.2	30	UN	[−100,100]	$f\left(x\right)=\overset{D}{\underset{i=1}{\sum }}{\left(\overset{i}{\underset{j=1}{\sum }}x_{j}\right)}^{2}$	*f*_*min*_=0
*f*_7_	Schwefel 2.22	30	UN	[−10,10]	$f\left(x\right)=\overset{D}{\underset{i=1}{\sum }}\left|x_{i}\right|+\overset{D}{\underset{i=1}{\prod }}\left|x_{i}\right|$	*f*_*min*_=0
*f*_8_	Schwefel 2.21	30		[−100,100]	$f\left(x\right)=\begin{array}{c} \max\\ i \end{array}\left\{\left|x_{i}\right|,1\leq i\leq D\right\}$	*f*_*min*_=0
*f*_9_	Bohachevsky1	2	MS	[−100,100]	$\begin{array}{c} f\left(x\right)=x_{1}^{2}+2x_{2}^{2}-0.3\cos\left(3\pi x_{1}\right)-0.4\cos\left(4\pi x_{2}\right)+0.7 \end{array}$	*f*_*min*_=0
*f*_10_	Bohachevsky2	2	MS	[−100,100]	$\begin{array}{c} f\left(x\right)=x_{1}^{2}+2x_{2}^{2}-0.3\cos\left(3\pi x_{1}\right)*\cos\left(4\pi x_{2}\right)+0.3 \end{array}$	*f*_*min*_=0
*f*_11_	Bohachevsky3	2	MS	[−100,100]	$\begin{array}{c} f\left(x\right)=x_{1}^{2}+2x_{2}^{2}-0.3\cos(\left(3\pi x_{1}\right)+\left(4\pi x_{2}\right))+0.3 \end{array}$	*f*_*min*_=0
*f*_12_	Booth	2	MS	[−10,10]	*f*(*x*) = (*x*_1_ + 2*x*_2_ − 7)^2^ + (2*x*_1_ + *x*_2_ − 5)^2^	*f*_*min*_=0
*f*_13_	Rastrigin	30	MS	[−5.12,5.12]	$f\left(x\right)=\sum_{i=1}^{D}\left[x_{i}^{2}-10\cos\left(2\pi x_{i}\right)+10\right]$	*f*_*min*_=0
*f*_14_	Schaffer	2	MN	[−100,100]	$f\left(x\right)=\frac{\mathrm{si}n^{2}\left(\sqrt{x_{1}^{2}+x_{2}^{2}}\right)-0.5}{{\left(1+0.001\left(x_{1}^{2}+x_{2}^{2}\right)\right)}^{2}}$	*f*_*min*_=0
*f*_15_	Six hump camel back	2	MN	[−5,5]	$f\left(x\right)=4x_{1}^{2}-2.1x_{1}^{4}+\frac{1}{3}x_{1}^{6}+x_{1}x_{2}-4x_{2}^{2}+4x_{2}^{4}$	*f*_*min*_ = − 1.03163
*f*_16_	Griewank	30	MN	[−600,600]	$f\left(x\right)=\frac{1}{4000}\overset{D}{\underset{i=1}{\sum }}x_{i}^{2}-\overset{D}{\underset{i=1}{\prod }}\cos\left(\frac{x_{i}}{\sqrt{i}}\right)+1$	*f*_*min*_=0
*f*_17_	Ackley	30	MN	[−32,32]	$\begin{array}{c} f\left(x\right)=-20\exp\left(-0.2\sqrt{\frac{1}{D}}\overset{D}{\underset{i=1}{\sum }}x_{i}^{2}\right)-\exp\left(\frac{1}{n}\overset{D}{\underset{i=1}{\sum }}\cos\left(2*\mathrm{pi}*x_{i}\right)\right)+20+e \end{array}$	*f*_*min*_=0
*f*_18_	Multimod	30	MS	[−10,10]	$f\left(x\right)=\overset{`D}{\underset{i=1}{\sum }}\left|x_{i}\right|\overset{D}{\underset{i=1}{\prod }}\left|x_{i}\right|$	*f*_*min*_=0
*f*_19_	Noncontinuous rastrigin	30	MS	[−5.12,5.12]	$\begin{array}{l} f\left(x\right)=\overset{D}{\underset{i=1}{\sum }}\left[y_{i}^{2}-10\cos\left(2\pi y_{i}\right)+10\right]\;\mathrm{Where}\;y_{i}=\left\{\begin{array}{c} x_{i}\;\left|x_{i}\right|<0.5\\ \frac{\mathrm{round}\left(2x_{i}\right)}{2}\;\left|x_{i}\right|\geq 0.5 \end{array}\;\right. \end{array}$	*f*_*min*_=0
*f*_20_	Weierstrass	30	MS	[−0.5, 0.5]	fx=∑i=1D∑k=0kmaxakcos2πbkxi+0.5−D∑k=0kmaxakcos2πbkxi+0.5,wherea=0.5,b=3,kmax=20	*f*_*min*_=0

In part2 of our experiments, attempts are made to compare our proposed approach with the recent variants of PSO as per (Zhan et al. 2009; Ratnaweera et al. 2004). The results of these variants are directly taken from (Zhan et al. 2009; Ratnaweera et al. 2004) and compared with OTLBO. In part3, the performance comparisons are made with the recent variants of DE as per (Zhan et al. 2009). The part4 of our experiments devote to the performance comparison of OTLBO with Artificial Bee Colony (ABC) variants as in (Alatas 2010; Zhu & Kwong 2010; Kang et al. 2011; Gao & Liu 2011). Readers may be intimated here that in all such above mentioned comparisons we have simulated OTLBO, **CoDE, EPSDE,** basic PSO, DE and TLBO of our own but gained results of other algorithms directly from the referred papers.

For comparing the speed of the algorithms, the first thing we require is a fair time measurement. The number of iterations or generations cannot be accepted as a time measure since the algorithms perform different amount of works in their inner loops, and they have different population sizes. Hence, we choose the number of *fitness function evaluations****(****FEs****)*** as a measure of computation time instead of generations or iterations. Since the algorithms are stochastic in nature, the results of two successive runs usually do not match. Hence, we have taken different independent runs (with different seeds of the random number generator) of each algorithm. Numbers of FEs for different algorithms which are compared with TLBO and OTLBO are taken as in (Zhan et al. 2009; Ratnaweera et al. 2004; Alatas 2010; Zhu & Kwong 2010; Kang et al. 12; Gao & Liu 2011). However, for TLBO and OTLBO we have chosen 4.0×10^4^ as maximum number of FEs. The exact numbers of FEs in which we get optimal results with TLBO and OTLBO are given in the Table 2.

**Table 2:** **No. of fitness evalution comparisons of PSO, DE, TLBO, OTLBO**

No.	Function		PSO	DE	TLBO	OTLBO
*f*_1_	Step	Mean	40,000	2.4833e+4	712	**520.1023**
		Std	0	753.6577	30.4450	**21.0012**
*f*_2_	Sphere	Mean	40,000	40,000	40,000	**28712**
		Std	0	0	0	**971.7807**
*f*_3_	SumSquares	Mean	40,000	40,000	40,000	**30124**
		Std	0	0	0	**150.0015**
*f*_4_	Quartic	Mean	40,000	40,000	40,000	**40,000**
		Std	0	0	0	**0**
*f*_5_	Zakharov	Mean	40,000	40,000	40,000	**2.9125e+04**
		Std	0	0	0	**150.1291**
*f*_6_	Schwefel 1.2	Mean	40,000	40,000	40,000	**31200**
		Std	0	0	0	**101.1902**
*f*_7_	Schwefel 2.22	Mean	40,000	40,000	40,000	**40,000**
		Std	0	0	0	**0**
*f*_8_	Schwefel 2.21	Mean	40,000	40,000	40,000	**40,000**
		Std	0	0	0	**0**
*f*_9_	Bohachevsky1	an	3200	4.1111e+03	1940	**1390**
		Std	51.6398	117.5409	79.8308	**30.2312**
*f*_10_	Bohachevsky2	Mean	3.1429e+03	4.2844e+003	2.0836e+03	**1290**
		Std	200.5150	201.8832	140.3219	**48.9012**
*f*_11_	Bohachevsky3	Mean	4945	7.7822e+03	2148	**1260**
		Std	168.1727	140.2739	51.4009	**39.1290**
*f*_12_	Booth	Mean	6420	1.2554e+004	3.4277e+03	**2.4519e+03**
		Std	18.3935	803.3543	121.4487	**150.5112**
*f*_13_	Rastrigin	Mean	40,000	40,000	4.4533e+03	**2.0912e+03**
		Std	0	0	544.6047	**77.1529**
*f*_14_	Schaffer	Mean	40,000	40,000	40,000	**4.0891e+03**
		Std	0	0	0	**149.9123**
*f*_15_	Six hump camel back	Mean	800	1.5556e+03	720	**430.2398**
		Std	99.2278	136.7738	33.0289	**23.0348**
*f*_16_	Griewank	Mean	40,000	40,000	2916	**1.7655e+003**
		Std	0	0	145.0686	**62.5381**
*f*_17_	Ackley	Mean	40,000	40,000	40,000	**40,000**
		Std	0	0	0	**0**
*f*_18_	Multimod	Mean	40,000	40,000	3488	**2239**
		Std	0	0	30.2715	**52.2319**
*f*_19_	Noncontinuous rastrigin	Mean	40,000	40,000	6.1891e+03	**2.2512e+03**
		Std	0	0	75.6887	**40.8082**
*f*_20_	Weierstrass	Mean	40,000	40,000	4.0178e+03	**2.2231e+03**
		Std	0	0	110.5696	**102.1123**

### Experiment 1: OTLBO vs. PSO, DE and TLBO

#### Parameter settings

In all experiments in this section, the values of the common parameters used in each algorithm such as population size and total evaluation number were chosen to be the same. Population size was 20 and the maximum number fitness function evaluation was 4.0×10^4^ for all functions. The other specific parameters of algorithms are given below:

*PSO Settings*: Cognitive and social components *c*_1_, *c*_2_, are constants that can be used to change the weighting between personal and population experience, respectively. In our experiments cognitive and social components were both set to 2. Inertia weight, which determines how the previous velocity of the particle influences the velocity in the next iteration, was 0.5.

*DE Settings:* In DE, F is a real constant which affects the differential variation between two Solutions and set to F = 0.5*(1+ rand (0, 1)) where rand (0, 1) is a uniformly distributed random number within the range [0, 1] in our experiments. Value of crossover rate, which controls the change of the diversity of the population, was chosen to be R = ( Rmax – Rmin) * (MAXIT–iter) / MAXIT where Rmax=1 and Rmin=0.5 are the maximum and minimum values of scale factor R, iter is the current iteration number and MAXIT is the maximum number of allowable iterations as recommended in (Swagatam & Ajith 2008).

*TLBO Settings:* For TLBO there is no such constant to set.

*OTLBO Settings:* For OTLBO, label Q= 6 is chosen emprically.

In Experiments 1, we compared the PSO, DE, TLBO and OTLBO algorithms on a large set of functions described in the previous section and are listed in Table 1. Each of the experiments in this section was repeated 30 times and it was terminated when it reached the maximum number of evaluations or when it reached the global minimum value with different random seeds and mean value and standard deviations of fitness value produced by the algorithms have been recorded in the Table 3 and at the same time mean value and standard deviations of no of fitness evaluation produced by the algorithms have been recorded in the Table 2.

**Table 3:** **Performance comparisons of PSO, DE, TLBO, OTLBO**

No.	Function	Global min/max		PSO	DE	TLBO	OTLBO
*f*_1_	Step	*f*_*min*_ = 0	Mean	203.3667	0	0	**0**
			Std	56.2296	0	0	**0**
*f*_2_	Sphere	*f*_*min*_ = 0	Mean	6.1515e-09	7.2140e-14	1.0425e-281	**0**
			Std	7.6615e-10	5.8941e-14	0	**0**
*f*_3_	SumSquares	*f*_*min*_ = 0	Mean	3.7584e-14	6.1535e-15	1.5997e-281	**0**
			Std	1.0019e-14	3.0555e-15	0	**0**
*f*_4_	Quartic	*f*_*min*_ = 0	Mean	1.9275	0.0253	2.3477e-04	**1.6911e-05**
			Std	1.4029	0.0075	1.7875e-04	**1.2211e-05**
*f*_5_	Zakharov	*f*_*min*_ = 0	Mean	141.0112	66.8339	1.4515e-281	**0**
			Std	40.7567	14.4046	0	**0**
*f*_6_	Schwefel 1.2	*f*_*min*_ = 0	Mean	9.3619e-08	5.3494e-13	2.6061e-270	**0**
			Std	6.6112e-08	4.6007e-13	0	**0**
*f*_7_	Schwefel 2.22	*f*_*min*_ = 0	Mean	9.3293	3.9546e-07	3.1583e-137	**2.1123e-221**
			Std	3.6619	1.9283e-07	1.7188e-137	**0**
*f*_8_	Schwefel 2.21	*f*_*min*_ = 0	Mean	60.9603	1.5340	4.3819e-136	**4.0091e-215**
			Std	4.0761	0.3900	1.5668e-136	**0**
*f*_9_	Bohachevsky1	*f*_*min*_ = 0	Mean	0	0	0	**0**
			Std	0	0	0	**0**
*f*_10_	Bohachevsky2	*f*_*min*_ = 0	Mean	0	0	0	**0**
			Std	0	0	0	**0**
*f*_11_	Bohachevsky3	*f*_*min*_ = 0	Mean	0	0	0	**0**
			Std	0	0	0	**0**
*f*_12_	Booth	*f*_*min*_ = 0	Mean	0	0	0	**0**
			Std	0	0	0	**0**
*f*_13_	Rastrigin	*f*_*min*_ = 0	Mean	76.2918	5.6344	0	**0**
			Std	17.1005	1.8667	0	**0**
*f*_14_	Schaffer	*f*_*min*_ = 0	Mean	0.0097	0.0029	0.0066	**0**
			Std	0.0025	0.0011	0.0045	**0**
*f*_15_	Six hump camel back	*f*_*min*_ = − 1.03163	Mean	−1.0316	−1.0316	−1.0316	**−1.0316**
			Std	0	0	0	**0**
*f*_16_	Griewank	*f*_*min*_ = 0	Mean	7.6291e-08	5.7841e-011	0	**0**
			Std	4.0012e-09	1.6914e-011	0	**0**
*f*_17_	Ackley	*f*_*min*_ = 0	Mean	14.0614	7.3814e-08	1.7171e-15	**1.1123e-15**
			Std	2.0125	3.0453e-08	1.5979e-15	**1.0021e-15**
*f*_18_	Multimod	*f*_*min*_ = 0	Mean	2.1994e-257	2.5678e-255	0	**0**
			Std	0	0	0	**0**
*f*_19_	Noncontinuous rastrigin	*f*_*min*_ = 0	Mean	100.3984	13.9237	0	**0**
			Std	28.7062	2.3146	0	**0**
*f*_20_	Weierstrass	*f*_*min*_ = 0	Mean	12.0447	1.5388e-05	0	**0**
			Std	2.6160	1.0139e-05	0	**0**

In order to analyze the results whether there is significance between the results of each algorithm, we performed t-test on pairs of algorithms which is quite popular among researchers in evolutionary computing (Das et al. 2009). In the Table 4 we report the statistical significance level of difference of the means of PSO and OTLBO algorithm, DE and OTLBO algorithm, TLBO and OTLBO algorithm. The t value is significant at a 0.05 level of significance by two tailed test. In table ‘+’ indicates the t value is significant’ means the difference of means is not statistical siginificant and ‘NA’ stands for Not applicable, covering cases for which the two algorithms achieve the same accuracy results. From the Table 4, we conclude that for 15 functions OTLBO technique is performing superior then PSO and in 5 functions they are similar in performance. While comparing OTLBO and DE, we can conclude OTLBO is significantly performing better in 14 functions and in 6 cases both are similar. However, between OTLBO and TLBO, the performance comparison is competitive. OTLBO is significant only for 9 functions but both are similar for 11 functions.

**Table 4:** **t value, significant at a 0.05 level of significance by two tailed test using Table** 3

Function no.	PSO/OTLBO	DE/OTLBO	TLBO/OTLBO
*f*_1_	**+**	NA	NA
*f*_2_	**+**	**+**	**+**
*f*_3_	**+**	**+**	**+**
*f*_4_	**+**	**+**	+
*f*_5_	**+**	**+**	**+**
*f*_6_	**+**	**+**	**+**
*f*_7_	**+**	**+**	**+**
*f*_8_	**+**	**+**	**+**
*f*_9_	NA	NA	NA
*f*_10_	NA	NA	NA
*f*_11_	NA	NA	NA
*f*_12_	NA	NA	NA
*f*_13_	**+**	**+**	NA
*f*_14_	**+**	**+**	+
*f*_15_	NA	**NA**	NA
*f*_16_	**+**	**+**	NA
*f*_17_	**+**	**+**	+
*f*_18_	**+**	**+**	NA
*f*_19_	**+**	+	NA
*f*_20_	**+**	+	NA

Table 5 reports the average ranking of optimization algorithms obtained by the Friedman’s test based on the performance using Table 3. The proposed OTLBO algorithm is ranked first, followed by TLBO, DE and PSO successively.

**Table 5:** **Average ranking of optimization algorithm based on the performance using Table** 3

Algorithm	PSO	DE	TLBO	OTLBO
Ranking	3.575	2.825	2.05	1.55

### Experiments 2: OTLBO vs. OEA, HPSO-TVAC, CLPSO and APSO

The experiments in this section constitute the comparison of the OTLBO algorithm versus OEA, HPSO-TVAC, CLPSO and APSO on 8 benchmarks described in (Zhan et al. 2009), where OEA uses the number of 3.0×10^5^ FEs and HPSO-TVAC, CLPSO and APSO use the number of 2.0×10^5^ FEs, where as OTLBO runs for 4.0×10^4^ FEs. The results of OEA, HPSO-TVAC, CLPSO and APSO are gained from (Zhan et al. 2009; Ratnaweera et al. 2004) directly. In the last column of Table 6 shows the siginificance level between best and second best algorithm using t test at a 0.05 level of significance by two tailed test. Note that here ‘+’ indicates the t value is significant means the difference of means is not statistical siginificant and ‘NA’ stands for Not applicable, covering cases for which the two algorithms achieve the same accuracy results. As can be seen from Table 6, OTLBO greatly outperforms OEA, HPSO-TVAC, CLPSO and APSO with better mean and standard deviation.

**Table 6:** **Performance comparisons OTLBO, OEA, HPSO-TVAC, CLPSO and APSO**

Function		OEA	HPSO-TVAC	CLPSO	APSO	OTLBO	Significant
Sphere	Mean	2.48e-30	3.38e-41	1.89e-19	1.45e-150	**0**	+
	Std	1.128e-29	8.50e-41	1.49e-19	5.73e-150	**0**	
Schwefel 2.22	Mean	2.068e-13	6.9e-23	1.01e-13	5.15e-84	**2.1123e-221**	+
	Std	2.440e-12	6.89e-23	6.54e-14	1.44e-83	**0**	
Schwefel 1.2	Mean	1.883e-09	2.89e-07	3.97e+02	1.0e-10	**0**	+
	Std	3.726e-9	2.97e-07	1.42e+02	2.13e-10	**0**	
Step	Mean	0	0	0	0	0	NA
	Std	0	0	0	0	0	
Rastrigin	Mean	5.430e-17	2.39	2.57e-11	5.8e-15	**0**	+
	Std	1.683e-16	3.71	6.64e-11	1.01e-14	**0**	
Noncontinous Rastrigin	Mean	N	1.83	0.167	4.14e-16	**0**	+
	Std	N	2.65	0.379	1.45e-15	**0**	
Ackley	Mean	5.336e-14	2.06e-10	2.01e-12	1.11e-14	**3.1123e-15**	+
	Std	2.945e-13	9.45e-10	9.22e-13	3.55e-15	**1.0021e-15**	
Griewank	Mean	1.317e-02	1.07e-02	6.45e-13	1.67e-02	**0**	+
	Std	1.561e-02	1.14e-02	2.07e-12	2.41e-02	**0**	

Table 7 reports the average ranking of optimization algorithms obtained by the Friedman’s test based on the performance using Table 6. The proposed OTLBO algorithm is ranked first, followed by APSO,OEA, HPSO, CLPSO successively.

**Table 7:** **Average ranking of optimization algorithm based on the performance using Table** 6

Algorithm	OEA	HPSO	CLPSO	APSO	OTLBO
Ranking	3.428571	3.71428	3.85714286	2.71428571	1.28571429

### Experiment 3: OTLBO vs. JADE, jDE, SaDE, CoDE, EPSDE

The experiments in this section constitute the comparison of the OTLBO algorithm versus SaDE, jDE, JADE, **CoDE** (Wang et al. 2011)**, EPSDE** (Mallipeddi et al. 2011) on 8 benchmark functions which are describe in (Zhan et al. 2009). The results of JADE, jDE and SaDE are gained from (Zhan et al. 2009) directly. In the last column of Table 8 shows the significance level between best and second best algorithm using t test at a 0.05 level of significance by two tailed test. Note that here ‘+’ indicates the t value is significant,’.’ means the difference of means is not statistical significant and ‘NA’ stands for Not applicable, covering cases for which the two algorithms achieve the same accuracy results It can be seen from Table 8 that OTLBO performs much better than these DE variants on almost all the functions.

**Table 8:** **Performance comparisons OTLBO, JADE, jDE ,SaDE,CoDE, EPSDE**

Function	FEs		SaDE	jDE	JADE	CoDE	EPSDE	OTLBO	Significant
Sphere	1.5× 10^5^	Mean	4.5e-20	2.5e-28	1.8e-60	1.12e-31	1.53e-85	**0**	+
		Std	1.9e-14	3.5e-28	8.4e-60	3.45-31	9.01e-86	**0**	
Schwefel 2.22	2.0× 10^5^	Mean	1.9e-14	1.5e-23	1.8e-25	1.22e-23	3.18e-54	**0**	+
		Std	1.1e-14	1.0e-23	8.8e-25	3.90e-23	3.11e-54	**0**	
Schwefel 1.2	5.0× 10^5^	Mean	9.0e-37	5.2e-14	5.7e-61	7.86e-31	4.81e-76	**0**	+
		Std	5.4e-36	1.1e-13	2.7e-60	1.86e-32	1.90e-76	**0**	
Step	1.0× 10^4^	Mean	9.3e+02	1.0e+03	2.9e+0	3.00e+00	0	**0**	NA
		Std	1.8e+02	2.2e+02	1.2e+0	1.90E+00	0	**0**	
Rastrigin	1.0× 10^5^	Mean	1.2e-03	1.5e-04	1.0e-04	1.21e-01	0	**0**	NA
		Std	6.5e-04	2.0e-04	6.0e-05	3.89e-02	0	**0**	
Schwefel 2.21	5.0× 10^5^.	Mean	7.4e-11	1.4e-15	8.2e-24	2.44e-27	1.94e-2	**0**	+
		Std	1.82e-10	1.0e-15	4.0e-23	1.89e-27	8.90e-4	**0**	
kley	5.0× 10^4^	Mean	2.7e-03	3.5e-04	8.2e-10	1.18e-04	5.36e-13	**2.78e-15**	+
		Std	5.1e-04	1.0e-04	6.9e-10	4.90e-04	4.77e-14	**1.56e-15**	
Griewank	5.0× 10^4^	Mean	7.8e-04	1.9e-05	9.9e-08	1.74e-07	0	**0**	NA
		Std	1.2e-03	5.8e-05	6.0e-07	2.33e-07	0	**0**	

Table 9 reports the average ranking of optimization algorithms obtained by the Friedman’s test based on the performance using Table 8. The proposed OTLBO algorithm is ranked first, followed by EPSDE, JADE, CoDE, jDE, SaDE successively.

**Table 9:** **Average ranking of optimization algorithm based on the performance using Table** 6

Algorithm	SaDE	jDE	JADE	CoDE	EPSDE	OTLBO
Ranking	5.375	4.875	3	4.25	2.3125	1.1875

### Experiment 4: OTLBO vs. CABC, GABC ,RABC and IABC

The experiments in this section constitute the comparison of the OTLBO algorithm versus CABC(Alatas 2010), GABC(Zhu & Kwong 2010), RABC(Kang 2011) and IABC(Gao & Liu 2011) on 8 benchmark functions. The parameters of the algorithms are identical to (Kang 2011). In the last column of Table 10 shows the significance level between best and second best algorithm using t test at a 0.05 level of significance by two tailed test. Note that here ‘+’ indicates the t value is significant means the difference of means is not statistical significant and ‘NA’ stands for Not applicable, covering cases for which the two algorithms achieve the same accuracy results The results, which have been summarized in Table 10, show that OTLBO performs much better in most cases than these ABC variants.

**Table 10:** **Performance comparisons of OTLBO, CABC, GABC ,RABC and IABC**

Function	FEs		CABC	GABC	RABC	IABC	OTLBO	Significant
Sphere	1.5× 10^5^	Mean	2.3e-40	3.6e-63	9.1e-61	5.34e-178	**0**	+
		Std	1.7e-40	5.7e-63	2.1e-60	0	**0**	
Schwefel 2.22	2.0× 10^5^	Mean	3.5e-30	4.8e-45	3.2e-74	8.82e-127	**0**	+
		Std	4.8e-30	1.4e-45	2.0e-73	3.49e-126	**0**	
Schwefel 1.2	5.0× 10^5^	Mean	8.4e+02	4.3e+02	2.9e-24	1.78e-65	**0**	+
		Std	9.1e+02	8.0e+02	1.5e-23	2.21e-65	**0**	
Step	1.0× 10^4^	Mean	0	0	0	0	**0**	NA
		Std	0	0	0	0	**0**	
Rastrigin	5.0× 10^4^	Mean	1.3e-00	1.5e-10	2.3e-02	0	**0**	+
		Std	2.7e-00	2.7e-10	5.1e-01	0	**0**	
Schwefel 2.21	5.0× 10^5^	Mean	6.1e-03	3.6e-06	2.8e-02	4.98e-38	**0**	+
		Std	5.7e-03	7.6e-07	1.7e-02	8.59e-38	**0**	
Ackley	5.0× 10^4^	Mean	1.0e-05	1.8e-09	9.6e-07	3.87e-14	**2.7812e-15**	+
		Std	2.4e-06	7.7e-10	8.3e-07	8.52e-15	**1.5611e-15**	
Griewank	5.0× 10^4^	Mean	1.2e-04	6.0e-13	8.7e-08	0	**0**	+
		Std	4.6e-04	7.7e-13	2.1e-08	0	**0**	

Table 11 reports the average ranking of optimization algorithms obtained by the Friedman’s test based on the performance using Table 10. The proposed OTLBO algorithm is ranked first, followed by IABC, GABC, RABC, CABC successively.

**Table 11:** **Average ranking of optimization algorithm based on the performance using Table** 7

Algorithm	CABC	GABC	RABC	IABC	OTLBO
Ranking	4.625	3.25	3.75	2.00	1.375

## Conclusions and further study

In this work orthogonal design approach is implemented to optimize global benchmark functions using basic Teaching-Learning based Optimization (TLBO). The proposed approach is known as Orthogonal TLBO (OTLBO).The benefit is derived in making the basic TLBO fasters with our proposed approach. Orthogonal design makes the search efficient in a large sample space to arrive at optimum solution. The paper discusses the fundamentals of orthogonal design and its framework in TLBO. The performance comparisons are done with TLBO and other evolutionary computation techniques like particle swarm optimization (PSO), Differential evolution (DE), artificial bee colony (ABC) and several of variants of these algorithms suggested by other researchers. From the results analysis it is evident that OTLBO outperforms all other approaches including basic TLBO for all benchmark functions investigated in our work. The efficiency of the proposed approach is compared with other algorithms in terms of number of function evaluations (FEs). We can conclude by saying that OTLBO is a very powerful approach of optimizing different types of problems which are separable, non-separable, unimodal and multimodal in providing quality optimum results in faster convergence time compared to very popular evolutionary techniques like PSO, DE, ABC and its variants. As a further research it remains to be seen how this adapts to multi-objective optimization problems and also some engineering applications from mechanical, chemical or data mining may be investigated.
